# Instagram fake profile detection using an ensemble learning method

**DOI:** 10.1038/s41598-025-03973-x

**Published:** 2025-07-21

**Authors:** Bharti Goyal, Nasib Singh Gill, Preeti Gulia, Noha Alduaiji, Piyush Kumar Shukla, Shreyas J

**Affiliations:** 1 Department of Computer Science & Applications, Maharshi Dayanand University, Rohtak, Haryana India; 2 Department of Computer Science, College of Computer and Information Sciences, Majmaah University, Al Majmaah, 11952 Saudi Arabia; 3 Department of Computer Science & Engineering, University Institute of Technology, Rajiv Gandhi Proudyogiki Vishwavidyalaya (State Technological University of Madhya Pradesh), Madhya Pradesh, Bhopal, 462033 India; 4 Department of Information Technology, Manipal Institute of Technology Bengaluru, Manipal Academy of Higher Education, Manipal, Karnataka 576104 India

**Keywords:** Fake profile, Instagram, XGBOOST, SMOTE, Random forest, Grid search CV, Scale_pos_weight, Evolution, Engineering, Mathematics and computing

## Abstract

Counterfeit accounts still pose a big problem for Instagram users. Trust is being eroded, and online security is being compromised as a result of these accounts’ constant contribution to Instagram’s spam, harmful information, and deceptive content problems. To find these profiles, we use a number of analytical parameters. Using machine learning is one of the main reasons for developing a model to effectively combat these false accounts. We investigate and provide a solution to the issue of Instagram’s ability to identify phony accounts in this research. An F1 score of 98%, a recall of 98%, a precision of 98.3%, and an accuracy of 98.24% are all achieved by the new, perfectly accurate model that is used in the proposed research. Our method combines scale_pos_weight optimization technique with XGBoost, SMOTE with balanced classes, and GridSearchCV to fine-tune key hyperparameters of Random Forest for fine-tuning purposes, therefore achieving this goal. This paper provides state-of-the-art methods for reducing the prevalence of false accounts, which will improve the efficiency and trustworthiness of identity verification systems used online. In this study, we provide an improved hybrid system with optimization that finds trends in phony accounts over time using adaptive discovery and strong analysis and class-balancing methods. In addition to improving online identity verification systems’ detection capabilities, this framework establishes a new standard for trust safeguarding via user trust and lays the groundwork for future breakthroughs in social media security.

## Introduction

Fake profiles have become a pervasive issue, this has also been a huge problem on Instagram lately, with the presence of fake profiles robbing users of trust in their own data and jeopardizing both security as well as the overall authenticity. These can be anything from spam-producing automated bots to tricked-out fakers. One of the main drivers to create those fake profiles is users looking for ways to artificially inflate their account by increasing visibility numbers and engagement levels, so it looks like they have a higher influence than reality. This is a trend that Instagram influencers are particularly guilty of, with many falsifying their online presence by buying followers, likes, comments etc. These activities land users and the platform in disrepute, not only putting them at risk of fraud, identity theft but also tarnishing the authentic legitimacy for influencers & monetization capability for platforms^1,2^. The presence of fake followers, as well as the purchasing of engagement by influencers, makes it difficult to quantify the influence and ROI for influencer movements. Therefore, it becomes difficult to establish a true and impactful marketing approach. There are some red flags which need to be considered in regard to the problem of fake accounts. For example, it is when followers, following are out of balance, or a huge number of followers is reported with extremely low engagement rates, or even posting patterns look suspicious. Vigilance with regard to such signs is quite necessary. Second, influencer publicity will be executed through specialized applications and tools that can help companies determine if the accounts recognized as influencers are legitimate or otherwise, thus preventing fraudulent activities from taking place^3,4^. The fake accounts, in return, use tactics such as buying followers, creating phony posts, and misrepresenting themselves to brands that invest in influencer marketing, resulting in economic losses and reputational loss. The large number of fake followers and purchased engagement makes it almost impossible to find out the exact influence of the influencer campaigns, thereby diluting the authenticity and impact of the campaign^5^. Apart from this, its further risks fraud, identity theft, breach of privacy, and low trust among users on the platform. These fake accounts also help in spreading false information. For this problem, researchers have developed a set of algorithms and approaches which deal with the issue. Most of these solutions leverage the vast quantity of unstructured data generated by social links to detect and identify fake accounts^6^. Machine learning algorithms have proven highly effective in detecting fake profiles, bots, and spam accounts on social media platforms. This research work is aimed at the development of a robust model using state-of-the-art machine learning techniques capable of effectively identifying fake profiles, thus making the online environment safer. This work, therefore, aims at establishing trust among its users, protecting authentic accounts, and further maintaining the integrity of the social media site, thus leading to better digital security and authenticity.

The main objective and motivation of this research lies with the rising number of false profiles on social media websites which presents serious challenges to information authenticity, user privacy, and digital security. With the advancements in machine learning, previous studies tend to emphasize single-modality approaches, while others using multimodality are unable to handle unbalance dataset, restricting detection accuracy. To fill this gap, our research introduces a hybrid model combining Random Forest, XGBoost, and SMOTE, utilizing both conventional ML and data resampling methods to improve classification accuracy. Our method particularly tackles imbalanced data distribution, a prevalent issue in fake profile detection, by using SMOTE for oversampling, so that minority class representations are well learned. Through parameter tuning of models and ensemble of predictions from Random Forest and XGBoost, we enhance generalizability and resilience. This study makes a contribution to the evolution of more adaptive and scalable solutions for fake account detection, addressing a crucial gap in the literature wherein hybrid machine learning models are yet to be adequately explored for this purpose.

This research is motivated by priority research questions to optimize fake profile detection on social media. In particular, we explore: (1) How does a hybrid model integrating Random Forest, XGBoost, and SMOTE enhance classification accuracy compared with conventional methods? (2) How does handling data imbalance influence model generalization and detection accuracy? (3) What are the best hyperparameter configurations for each model to optimize detection reliability? By structured experimentation and comparison, we strive to present a strong and scalable solution that enhances detection precision without the constraints of current single-model solutions. Our research advanced hybrid machine learning methods for social media security to effectively detect fraudulent accounts."

This research goals to accomplish the following major objectives:

1. Investigate the frequency of fake Instagram accounts.
2. Propose active fake profile detection approaches.
3. Guide imminent research and platform strategies.

The organisation of the rest part of paper is as: Section"Random forest optimization"provides a literature analysis on fake profile detection, critiquing the several approaches proposed in early studies. Section"XGBoost Optimization"outlines the research methodology employed for detecting fake profiles. Section"Model fusion"represents the results, offering a discussion in context of prior research and the problem at hand, while emphasizing the strengths and weaknesses of the proposed approach. Section"Training & validation analysis"encapsulates the main conclusions of the article, emphasising the importance of identifying fake profiles and the possible impacts for municipal, platforms, and society at large.

## Related work

In modern era, fake profile detection has attracted considerable academic attention. Machine learning has become a widely used approach for this task, encompassing data collection, feature extraction, and ordering to assess the authenticity of profiles. The most critical issue involves the removal of spam accounts and the ready availability of strong datasets to evaluate. Studies indicate that, by using machine learning algorithms, fraudulent profiles can be easily identified, and commonly measured performance is accuracy, F1-score, and recall. Overall, advancements in machine learning offer promising solutions in identifying fake profiles on such social platforms.

### Fake profile impact

Fake accounts cover all forms of online business and communication. These are a grave threat to authenticity and consistency in the sites’ information. They present an atmosphere where it is so hard for consumers to recognize fact from fiction, eventually spreading myths. The detection of fake social media accounts is crucial as there may be consequences in relation to the platform, its users, and the entire society. The sudden increase of fake accounts has also become a concern either as created by individuals, bots, or automated systems. There are several concerns such as spreading false information, engaging in illegal activities such as character burglary and phishing, and rumours, which may create rigidities and divergences in society^7^. Meanwhile, identification of spurious accounts is a top priority for consumers because it ensures reduced risk of fraud, identity theft, and their respective breaches of privacy. Once these spurious accounts are identified, users can disable them to avoid being misled by the erroneous evidence, avoid the situation of cyberbullying, and have a safe online space for communication and data discussion. It is necessary to detect fake profiles on social networking sites to preserve authenticity, confidence, and user rendezvous, ensuring continuous use of the platform. Most social sites, including Facebook, Instagram, and Twitter, rely on authentic user contacts to provide positive involvement and maintain their status as credible sources of information and communication. By successfully detecting and eliminating fraudulent accounts, platforms may boost user trust, prevent the spread of false information, and preserve their integrity as social services. The implications of identifying fake profiles in society are far-reaching and significant. Fake accounts pay to the spread of harmful content, and misinformation, which can influence civic belief, generate conflicts, and erode trust in online civilizations. Society’s advance detection methods can help check malpractices through fake accounts and protect vulnerable individuals from such things, creating a safer and more trustworthy online environment for social communication and data sharing^8^.

Thus, the significance of detecting fraudulent accounts on public websites, like Instagram, would be composed of important elements like the influence on users, the influence on the platforms, and the influence on society as large. Here are the whys and wherefores of why detecting fake profiles is so important:

1. Online environment misrepresentation: By increasing a person’s or group’s follower count, fake accounts can significantly distort the online landscape and increase the popularity of the individual or group^9^. Additionally, consumers may be exposed to misleading interactions and content through dishonest accounts, which could negatively impact their experience. By detecting and deleting duplicitous accounts, social mass media can increase user involvement and create a more authentic and meaningful online environment^10^.
2. Destruction of confidence: The presence of spam accounts can lead to an eroding self-confidence in online environment, since the users are more likely to question the credibility of the information they encounter. This scepticism may eventually result in a substantial loss of faith^11^. Social media networks’ legitimacy may be threatened by accounts, which would have a damaging effect on users’ confidence and reputations. Trustworthiness has been an increasingly important element in shaping public discourse, and data distribution is important for the identification and removal of spurious accounts^12^.
3. Amplification of false information: False stories can be used to spread information and sway public opinion on a variety of subjects, including important concerns and health information^13^. Fake accounts are used to influence civic belief, change the work atmosphere, and destroy confidence in social networks. By identifying and eliminating fraudulent accounts, the internet becomes a more trustworthy medium by reducing the dissemination of misleading information^14^.
4. Threats to User-Privacy and Safety: Fake-account activities can perform some harmful illicit tasks, including spoofs, phishing, or spamming, thereby potentially posing threats to the private and safe use of other users’ accounts in social networks. Even fake user photographs, ends up propagating misinformation. The severe issue of image forgeries needs to be tackled^15^. Such threats from malicious account-related fraudulent data can be reduced or completely halted by implementing efficient detection schemes that detect and delete those related accounts into the network, protecting users accordingly^16^.
5. Problems for social marketing: It makes officialdoms that hugely rely on social media very challenging to deal with issues of false accounts. As such, these entities end up having a significant proportion of their bull’s eye observers being slow or fraudulent^17^. Fraudulent profiles can create challenges for businesses that utilize social media for selling and appointment, as they may inadvertently target numerous fake or inactive accounts. Implementing effective detection technologies can assist these entities in achieving their legitimate objectives on social media by ensuring genuine participation and interactions^18^.

To address these issue, social media platforms have implemented various measures, including authentication systems for prominence users and algorithms designed to detect and remove suspicious accounts. However, this problem remains persistent, and the ongoing growth of new methods to identify and block fraudulent accounts remains a urgency for both researchers and platform operatives^19^.

### Detection approaches

Several ways to detect spammers or fake profiles on social networking sites have been put forward. Most of them apply the machine learning algorithms on huge amounts of data generated from such social sites^20^. The detection of spurious profiles is essentially hybridizing multiple machine learning techniques. This approach posed a proposed hybrid model to correctly identify deceptive accounts. One effective method is by using a machine learning algorithm that distinguishes between a spurious and a genuine account. Experimental results prove that the efficiency of distinguishing between genuine and fraudulent accounts is highly possible and feasible through machine learning. Another discussed method is a hybrid approach in detecting fake Twitter accounts based on a solution that implements a Chrome extension. Such a framework bases its evaluation on the possible parameters provided by the extension to figure out the extent of falsification of given profiles. The articles are also inclined toward the unstructured social network data which can lead to the establishment of algorithms and techniques for profiling fake profile identification. This data will, therefore, be key to the realization of the patterns and attributes commonly assigned to the rogue accounts. In general, social media data analysis stands at the heart of proper detection and response to growing fake accounts across the systems^21^. Various methodologies used for the identification of fake profiles include:

1. Hybrid model: A research paper developed an Artificial Intelligence hybrid model designed to detect malicious fake profiles across various social networks. This hybrid model-based advanced machine learning technique approach for fraudulent account identification was able to successfully identify fraudulent accounts through the entire investigation of this proposed method^22^.
2. Supervised algorithms: A research article suggested that for the detection of spurious profiles, a few supervised algorithms could be applied. It was observed that in a profile acceptability evaluation the approach used was the basic visual appearance approach. It focuses on numerical data and avoids inclusion of categorical features in refinement of analysis. The effective methodology proposed by this study ensured the identification of fake-account, overcoming the previous limitation of failing to identify a fraudulent user^23^.
3. Support vector machine: A study^24^ introduced a classification scheme to classify spam and legitimate profiles that used Support-Vector-Machine for classification. The basic strategy used was: first step of profile selection; identification of the features, important to classification; inputs and a trained SVM classifier then it decided whether a particular profile was genuine or a fake one. Table 1 displays some of the earlier research conducted in this area, including:

**Table 1:** Previous work analysis.

Reference	Techniques used	Brief discussion
C. Lin et al.^25^	Machine learning	A shilling-attack-model, Leg-UP is proposed to form untraceable fake profiles
K. Zarei et al.^26^	Deep neural network	Real content, fan-generated content, and bot-generated content were all successfully identified as phony profiles
M. Mohammadrezaei et al.^27^	PCA, SMOTE,	Friend similarity is casted for fake account detection
P. Shahane et al.^28^	Deep convolutional neural networks, random forest	Twitter fake profiles are detected
S. Siva Rama Krishna et al.^29^	KNN	Profile cloning is detected on online social networks
P. Sowmya et al.^30^	Distance measure and classification algorithms	Fake-clone accounts on twitter are detected
S. Terumalasetti et al.^31^	deep learning	Fake bot account detection framework is presented to analyze multimodal features: Visual content, temporal activity, and network interactions
L. Li et al.^32^	Machine learning	2019 Climate extinction rebellion protests is observed and it is found that social bots should be monitored as new technology makes identifying bots from people harder

#### A detailed amount of work done previously is as

Y. Perwej et al.^33^ found out spam and automated Instagram account which creates fake interactions in the process. Seven machine learning classifier techniques showed correlation with the identified process: J48, Random Forest, K-Nearest Neighbors (KNN), Neural Networks, logistic regression, and the Naive Bayes algorithm following the application of correlation and singular value decomposition (SVD). It was found to be a success rate 99.43% based on correlation combined with a neural network algorithm. F. C. Akyon et al.^34^ identified spam account on instagram using SVM, logistic regression, Naive Bayes, and neural networks. B. Goyal et al.^35^ conducted comparative study on Instagram by using two different datasets with various techniques and concluded finally that performance of the model depends upon the dataset being used and techniques applied. Using machine learning approaches, A. S. Fathima et al.^36^ detect the establishment of phony profiles in several social media networks. The study is strengthened with a large-scale database comprising various parameters of different user behavior. Stringent data preparation methods have followed in this research work wherein the feature scaling was achieved, and the entire set of data was divided into sub-training and testing datasets; A Neural-Network model had been used. Results from experiments proved that the model succeeded in correctly detecting fake profiles based on a very detailed analysis of the behavioral patterns of fake accounts.E. Y. Güven et al.^37^ proposed a system for classifying public Instagram accounts by examining comments, profile images, biographies, and posts shared by users with business accounts. The process began with the development of a crawler to collect data, which was then anonymized. The final model achieved a 95% accuracy, enabling proof of identity of diverse business-accounts on Instagram. F. Bertini et al.^38^ discovered image-watermarking for fake-profile-detection. M. Egele et al.^39^detected fake accounts using COMPA in protuberant officialdoms. Z. Wu. Et al^40^. safeguarded users’ private information within recommender systems. Z. Qu et al.^41^detected Sybil using MUSH on user-reviewed-social-networks. A study by N. S. G. Bharti et el^42^. utilized various approaches, including SVM-RF-NB, to detect fake-accounts athwart societal platforms. The research involved collecting raw data, extracting relevant features to identify fake profiles. Among the methods tested, the study concluded that SVM outperformed the other techniques in noticing fraudulent profiles. P. Krishnan et al.^43^ proposed a finite-automata-based detection method that utilized Deterministic Finite Automata, pattern-matching, and regular-expressions. While this approach demonstrated promising results in identifying fake profiles, it faced a limitation: The size of the regular expressions increased as the number of social network friend groups grew, which could impact the method’s efficiency. K. Zarei et al.^44^ focused on detecting mimics on Instagram by using Natural Language Processing (NLP) to analyse comments. The researchers effectively identified impersonators involved in both active–passive engagements, especially in cases related to politicians, news-agencies, and sports-figures. J. Schler et al.^45^ developed feature sets for both pre-election and post-election periods during the Israel elections, and employed numerous algorithms. By analysis the researchers successfully distinguished between bot accounts and human users. Z. Yang et al.^46^ used BiGRU and BERT algorithms to spot social spam bots on the beses of temporal content. N. S. Gill et al.^47^ used well conserved user data in cybernetic world via machine learning methods. T. S. Nivas et al.^48^ proposed a study introducing a novel method named ADB-CB, designed to enhance the uncovering of fake Instagram accounts. This approach utilizes three discrete ML algorithms including Adaboost, Catboost, and Extra-Trees classifier along with advanced methods for feature selection and dimensionality decrease. P. Durga et al.^49^ utilized logistic regression, k-nearest neighbor, and DT algorithms in emerging a supervised ML ning model. There were two different tests used in the study, and among the three models developed, the DL worked best at a 96% success rate for accuracy in comparison of models.

With this, in the context of hybrid DL methods, a number of studies have investigated the mixture of various neural network structural design to enhance Arabic text classification. Abdulghani and Abdullah^50^ suggested a CNN–LSTM hybrid model that used mutual information for feature selection, which provided improved classification accuracy on Arabic datasets. Zahidi et al.^51^ also created a CNN–LSTM hybrid model for Arabic soppiness examination, using word embedding models to extract semantic features in text data. Additionally, the use of transformer models for Arabic dialect identification has been researched. For example, a paper in PMC^52^ examined the implementation of stacked transformer models for the improvement of Arabic dialect identification and showed the effectiveness of such models in managing dialectical differences. Furthermore, Chowdhury et al.^53^ also highlighted the need for diversifying training data for transformer models to enhance Arabic text categorization, underlining the advantage of employing varied datasets in maximizing model performance.

Collectively, these studies emphasize the growing curiosity and progress in applying advanced transformer-based and hybrid DL models to Arabic text organization, make a speech the unique tasks postured by the language’s complex morphology and dialectal diversity.

These studies cover a wide range of methodologies, from blockchain, feeling examination, DL, ML, and behavioral modeling addressing various issues in cybersecurity, crime detection, and social media analysis. The literature review points out the fact that many earlier approaches focused narrowly on particular features or relied upon outdated methods that make them inefficient against the changing tactics used by the people who are creating these fake accounts. Further, such approaches are less adaptable in responding to new forms of fraudulent behaviour and, thus, lose their effectiveness in the long run. Since traditional ML models RF and XGBoost have been predominantly used in previous literature on fake profile detection; however, they are known to be inefficient for class imbalance and feature selection. SMOTE has been used for handling data imbalance, but resampling sometimes may introduce noise that degrades performance. Moreover, hyperparameter optimization techniques GridSearchCV have not been applied to most studies, which again results in suboptimal configurations of the model. Thus, to overcome the shortcomings of the aforementioned, our proposed method is an optimized hybrid approach that combines Random Forest with XGBoost with SMOTE for the balanced data distribution. By Hyperparameter Tuning, it adjusts `max_depth`, `min_samples_split` and `n_estimators`, for Random Forest and `scale_pos_weight`, `learning_rate`, and `n_estimators` for XGBoost to increase performance without overfitting. To make it different, our methodology essentially optimizes combination from both sides, thereby outperforming each other in having a better level of generalization. Moreover, we present our study with respect to the current studies to introduce a comparative improvement with previous knowledge to show where we gain this optimization, bringing higher classification accuracies and much robustness due to imbalances.

Since detection of fake profiles has been an imperative field of research in internet security, and there have been various studies that use ML^54^ and DL-based methods. Most conventional approaches have focused on manually designed features including user behaviour patterns, language clues, and social network topology. The drawback to these methods, however, is that they struggle to handle intelligent fake profiles with the ability to mimic human interactions.

New developments in ensemble learning and DL have greatly enhanced the recognition of spurious accounts. Experiments based on RF and XGBoost (XGB)^55^ classifiers have proven to perform well in dealing with structured data. As an example, work that leveraged XGBoost has exhibited enhanced classification performance as a result of the capability of capturing complex feature interactions. Likewise, SMOTE has been extensively used to handle the class imbalance issue in fake account detection tasks to improve model generalizability^56^. Also, hybrid approaches based on combining various classifiers have been introduced to further enhance detection efficiency. Some techniques involve combining Convolutional Neural Networks (CNNs)^57^to analyse profile pictures, whereas others use Recurrent Neural Networks (RNNs) or LSTMs^58^in order to model temporal patterns within user behaviour. Additionally, graph-based methods involving analysing user interactions and relationships have been investigated in order to enhance the detection of synchronized bot networks^59^.

Our research advances these previous methods by combining Random Forest and XGBoost within a hybrid method, tuned for hyperparameters via GridSearchCV. We also use SMOTE to maintain balanced training and avoid the impacts of skewed datasets. Through these methods combined, our research increases classification accuracy and resilience, solving for the flaws in one-model strategies.

Although transformer-based models like BERT^60^ and deep learning models like LSTM^61^ have exposed notable presentation in numerous NLP jobs, our research is more concerned with the efficacy of user metadata features in identifying fake profiles. The decision to employ conventional ML models—namely Random Forest, XGBoost, and Logistic Regression—was guided by two primary considerations. First, the dataset used in this study is structured and metadata-driven, comprising numerical and categorical features such as the number of followers, biography length, and the presence of a profile image. These characteristics lend themselves more effectively to traditional algorithms, with minimal reliance on raw textual data. Second, advanced models like BERT naturally require substantial amounts of labeled textual data, significant computational assets, and extensive fine-tuning, all of which were beyond the practical scope of this investigation. Additionally, integrating models such as BERT or LSTM would have necessitated substantial changes to the data preprocessing pipeline and model architecture, diverging from the study’s focus on metadata-based fake profile detection.

The primary gap in fake profile detection research lies in the limitations of existing methods, which often struggle with class imbalance, ineffective feature utilization, and suboptimal performance due to a lack of hyperparameter tuning. Many prior studies rely on single-model approaches, such as Random Forest or XGBoost, without leveraging their complementary strengths. Additionally, while SMOTE has been used for class balancing, improper oversampling can introduce noise and hinder model generalization.

The literature rarely explores optimized hybrid frameworks that combine multiple models with fine-tuned parameters for improved classification accuracy. Our study addresses these gaps by integrating an optimized combination of Random Forest and XGBoost, incorporating SMOTE for data balancing, and applying hyperparameter tuning to enhance performance. We systematically analyze the impact of each optimization step and compare our results with existing studies to afford a more robust and accurate solution for fake profile finding.

We achieved high accuracy by enhancing our model’s adaptability to the energetic nature of fake accounts. Our approach makes sure robustness against evolving techniques used by fake account creators, whereas previous studies have struggled with these challenges, particularly on Instagram.

## Methodology

This section, describes the research methodology smashed in detail, including the ways for data gathering, the extensive pre-processing processes, and the amalgamation of hybrid feature learning techniques using XGBoost, SMOTE, and Random Forest. Within this section, the hybridisation process is dissected in further depth, culminating in the forecasting of Accuracy, Precision, Recall, and F1-Score.

### Data pre-processing and feature selection

**Fig. 1:**
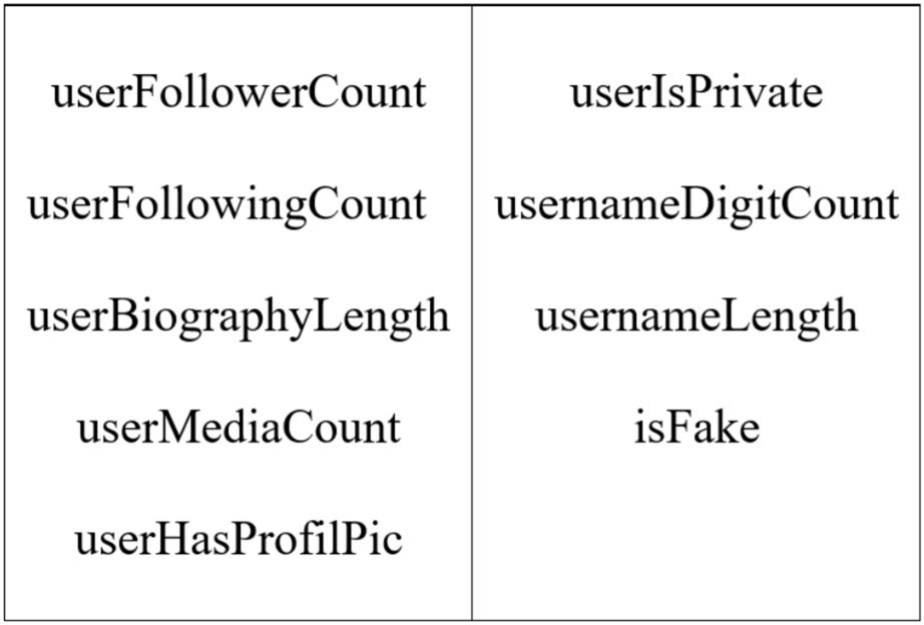
Dataset features.

The dataset used is carefully selected to notice of fake profiles on Instagram^34^. The data collection manner complexes an open-source library containing tagged Instagram accounts separated actual and fake profiles for data collecting. The dataset consists of 200 fake accounts and 994 real accounts, respectively with eight account authenticity features. A new feature “isFake” is added for binary classification of real and fake accounts. Since the dataset selected for this research is nominal, Binary features including userHasProfilePic and userIsPrivate were important, as real users often had profile pictures and public profiles. usernameDigitCount and usernameLength also helped differentiate accounts based on naming patterns, with fake profiles often having more digits or unusual lengths. Additionally, userBiographyLength served as an indicator of authenticity, as legitimate accounts typically have more detailed bios compared to fake ones. Features selected considered the behavior of users, including userFollowerCount, userFollowingCount, userBiographyLength, userMediaCount, usernameLength, and isFake (target variable). Furthermore, normalization operations are used to transform numerical features to a similar range so that the influence of different scales would not distort the learning outcome. This was a highly imbalanced dataset, as fake profiles significantly outnumbered the genuine ones. SMOTE synthesis of the class was thus necessary to stability the dataset. SMOTE synthesized samples of the smaller class augmented the representation of this class and improved the model’s aptitude to classify fake accounts. Subsequently, the pre-processed dataset was split into preparation and test sets with 80:20, safeguarding that all class distributions are followed over subsets. Therefore, by these pre-processing stages, the dataset reliability, representativeness, and readiness for training high-advanced machine learning models are guaranteed. Thus, accurate robust classifications are ensured. The dataset features are exposed in Fig. 1 as:

### Techniques used

The proposed fake profile detection approach combines XGBoost, SMOTE, and Random Forest to ensure robust and accurate classification. XGBoost is utilized for its efficiency in building decision trees iteratively, correcting previous errors, and handling large datasets with built-in regularization to prevent overfitting. SMOTE ensures class imbalance through the production of artificial samples for the minority so that the model learns it with efficiency. The techniques by Random Forest handle the building of decision trees to further come up with the overall best diversified pattern that stops overfitting. In these, the methods build the flexible system with balance required for the finding of fake profile identification on Instagram.

#### XGBOOST

Extremely fast gradient boosting machine: extreme gradient boosting is one such ML algorithm applied majorly for organization and reversion jobs. It is a part of the family of gradient boosting machines, hence improving the predictability because decision trees are created progressively in an attempt to solve errors made by the previously chosen models. XGBoost has a huge emphasis on speed and performance that gives good results in very big and complex datasets. XGBoost is widely applied in a variety of fields, such as fake profile detection, fraud detection, etc. It identifies suspicious activities by recognizing patterns in large datasets of transactions, and it is also widely used in building recommendation systems for e-commerce and streaming platforms where predicting user preferences is central. In this respect, XGBoost is used because of its strength, speed, and flexibility regarding handling structured and unstructured data.

#### SMOTE

SMOTE is one of the major algorithms in ML. Its primary objective is to tackle class imbalance and improve balance in a dataset by providing synthetic samples for the minor class that is underrepresented. The difference between over-sampling techniques and SMOTE lies in the fact that instead of copying minority instances, it generates new unique instances with the help of interpolation of existing minority samples. This method makes the models learn more about the minority class and thereby enhances their presentation in such parts as fraud finding, medical diagnostics, and text classification, where the presence of an imbalanced dataset is usual. SMOTE reduces bias concerning the common class, thereby enhancing model robustness and simplification capability.

#### Random forest

It is a heavy-duty ML algorithm and generally discarded for classification assignments, such as identifying rogue Instagram profiles. It learns by creating many decision trees at training time and their combined prophecies enable even more accurate and resilient organizations. Starting with library work such as using libraries such as scikit-learn in Python, it’s initiated with data training extracting important features from the Instagram data. Decision trees are constructed through Random Forest by choosing subsets of random features, reducing overfitting and, hence improving the generalization. Different feature combinations made by the decision trees are aggregated into predictions made by the classifier to classify an account as real or fake. Performance is also evaluated using assessment techniques including hyperparameter tuning and cross-validation.

### Experiment work

As experimental work in proposed model fine-tuning included hyperparameter tuning and early stopping of the Random Forest as well as the XGBoost classifiers are performed. In order to correct class imbalance within the dataset, SMOTE is used, which ensured the model is exposed to a balanced number of both real and fake accounts prior to dividing the dataset into preparation and justification sets. For RF, GridSearchCV-based hyperparameter tuning with three-fold cross-validation to tune important parameters including the number of estimators tree depth (`max_depth`), (`n_estimators`), and the minimum number of samples needed to split a node (`min_samples_split`) are carried. This allows to choose the best Random Forest setting. For XGBoost, also the model is optimized by setting `scale_pos_weight` to mitigate class imbalance, enabling early stopping with `early_stopping_rounds = 10` to avoid overfitting, and using the evaluation metric of `logloss`. The combined model is next optimized by combining predictions from the two classifiers based on a weighted averaging technique, where we adjusted dynamically at each epoch the contribution of the probability scores from each model. In order to guarantee the consistency of solution, we assessed model performance on classification reports, accuracy scores, confusion matrices, and training-validation loss plots.

## Proposed model

In this section, the workflow included processes related to data-retrieval, merging, pre-processing, and then using algorithms, which were especially important given the use of Instagram datasets. Furthermore, the underlying model is founded on existing learning algorithms RF and XGBoost, the nature of this innovation is in applying a hybrid approach combined with optimization techniques to effectively enhance fake-profile detection. That is, such a study would not just pick up these models but strategically deploy them with different optimization techniques. First, GridSearchCV-based hyperparameter tuning was conducted to fine-tune Random Forest, ensuring optimal depth and tree count to balance performance and generalization. Second, rather than oversampling, XGBoost was optimized using scale_pos_weight to handle class imbalance effectively, which prevents data duplication and reduces computational overhead. In addition, the use of weighted voting by combining the Random Forest and XGBoost prediction further increased robustness based on both model strengths, specifically Random Forest, which easily manages structured data and XGBoost, known for its ability in gradient boosting in improving the precision of the classifier. Feature engineering was performed in the dataset; the process also involved a strict evaluation by making use of cross-validation for verifying consistency. The final model resulted in 98.24% accuracy against individual classifiers. Although the existing algorithms were used, novelty is added by introducing an optimized hybrid framework with enhanced class balancing techniques, feature extraction strategies, and improved decision-making that leads to more reliable and interpretable fake profile detection.

Mathematical Modelling for Fake Profile Detection is shown step by step as:

1. Data pre-processing & class imbalance handling

1 $${X}_{\text{new}}={X}_{i}+\uplambda \left({X}_{j}-{X}_{i}\right),\hspace{1em}\uplambda \sim U\left(\text{0,1}\right)$$

2 $${D}_{\text{train}},{D}_{\text{val}}={\text{train}}\text{\_}{\text{test}}\text{\_}{\text{split}}\left({D}_{\text{SMOTE}},0.2\right)$$

Equation (1) represents the data pre-processing where 0 represents real and 1 represents fake accounts and in Eq. (2) data is divided in train and test sets with 80:20 ratios and SMOTE is applied to handle unbalanced data in the dataset.

2. Random forest optimization

3 $$\widehat{y}=\frac{1}{T}{\sum }_{t=1}^{T}{h}_{t}\left(X\right)$$

Hyperparameters:

$$T\in \{\text{50,100}\},$$

$${\text{max}}\text{\_}{\text{depth}}\in \{None,\text{10,20}\},$$

$${\text{min}}\text{\_}{\text{samples}}\text{\_}{\text{split}}\in \{\text{2,5}\}$$

Optimized model:

4 $$\widehat{{h}_{\text{RF}}}\left(X\right)={\text{argmin}}_{h}{\sum }_{i=1}^{N}\mathcal{L}\left({y}_{i},h\left({X}_{i}\right)\right)$$

Using Eqs. (3) and (4) Random Forest is optimized where in Eq. (3) hyperparameter tuning is performed and Eq. (4) shows optimized Random Forest.

3. XGBoost optimization

5 $$\widehat{{y}^{\left(t\right)}}=\widehat{{y}^{\left(t-1\right)}}+\upeta {f}_{t}\left(X\right)$$

Objective function:

6 $$\mathcal{L}\left(\uptheta \right)={\sum }_{i=1}^{N}L\left({y}_{i},\widehat{{y}_{i}}\right)+{\sum }_{t=1}^{T}\Omega \left({f}_{t}\right)$$

Hyperparameters:

$$w=\frac{{N}_{\text{neg}}}{{N}_{\text{pos}}},$$

$$T\in \{\text{50,100,150}\},$$

$$\upeta \in \{\text{0.01,0.05,0.1}\}$$

XGBoost optimization is performed in step 3 where Eq. (6) represents the objective function.

4. Model fusion

$${P}_{\text{RF}}\left(X\right)=\widehat{{h}_{\text{RF}}}\left(X\right),\hspace{1em}{P}_{\text{XGB}}\left(X\right)=\widehat{{h}_{\text{XGB}}}\left(X\right)$$

7 $${P}_{\text{final}}\left(X\right)=\frac{{P}_{\text{RF}}\left(X\right)+{P}_{\text{XGB}}\left(X\right)}{2}$$

8 $$\widehat{{y}_{\text{final}}}=1\left({P}_{\text{final}}\left(X\right)\ge 0.5\right)$$

Finally, Random Forest and XGBoost fusion is applied as shown in Eq. (7) and then final values are predicted as shown in Eq. (8).

5. Training & validation analysis

9 $${\text{Acc}}_{\text{train}}^{\left(e\right)}=\frac{{\sum }_{i=1}^{N}1\left(\widehat{{y}_{i}^{\left(e\right)}}={y}_{i}\right)}{N}$$

10 $${\text{Loss}}_{\text{train}}^{\left(e\right)}=\mathcal{L}\left({y}_{\text{train}},\widehat{{y}_{\text{train}}^{\left(e\right)}}\right)$$

Training Accuracy and Loss are represented by Eq. (9) and (10) respectively.

Final accuracy:

11 $${\text{Accuracy}}_{\text{final}}=\frac{{\sum }_{i=1}^{N}{1\!\,\!\!1}\left(\widehat{{y}_{i}}={y}_{i}\right)}{N}$$

**Fig. 2:**
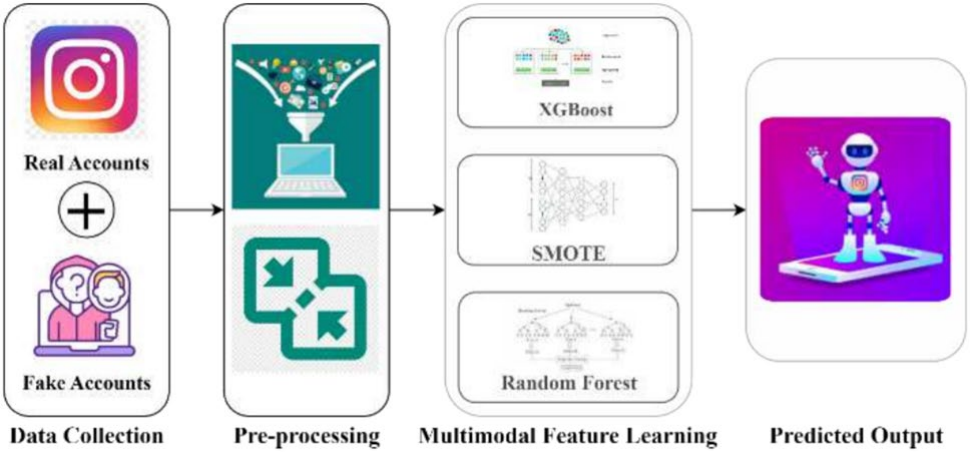
InstaFake architecture.

In this section, the research process is minutely designed. It first deals with the collection and pre-processing of the dataset, followed by an advanced model configuration that involves the state-of-the-art algorithms with comprehensive parameter tuning. It runs systematic tests over different scenarios to learn more about the behaviour of the model and gain deep insights into its resilience as well as adaptability. In fact, results are more appreciable to give perceptions for assets and limitations of a model supported by detailed analyses. Figure 2 illustrates the proposed hybrid architecture.

A step-by-step procedure in mathematical form is shown by algorithm as:

### Algorithm: Instafake

*Let X* ∈ R^*n*×*m*^ represents the feature matrix, where *n, and m are* the number of samples and features respectively,

*y* ∈ {0*,* 1}^*n*^ where 0 indicates real, and 1 indicates fake account.

12 $$X^{\prime } , \, y^{\prime } = {\text{ SMOTE }}\left( {X, \, y} \right)$$

In Eq. (12) SMOTE is applied to dataset to balance the dataset, where *X*^′^ and *y*^′^ are the balanced feature matrix and label vector.

13 $$X_{{{\text{train}}}} , \, X_{{{\text{val}}}} , \, y_{{{\text{train}}}} , \, y_{{{\text{val}}}} = {\text{ train test split }}\left( {X^{\prime } , \, y^{\prime } } \right)$$

In Eq. (13) dataset is divided into train and test sets, where* X*_train_*, y*_train_ are the training data and *X*_val_*, y*_val_ are the validation data.

14 $$T_{i} :h_{i} \left( X \right) \, = {\text{ Tree}}_{i} \left( X \right)$$

Eq. (14) represents the classifier selected.15

The final Random Forest model prediction for *k* trees is represented by Eq. (15).16

In Eq. (16)* F*_*t*_ is the prediction at round *t*, *η* is the learning rate, and *g*_*i*_ (*X*) are the gradient-based updates.

After *T* rounds (epochs), the final prediction is represented by Eq. (18). With Eq. (17) representing XGBoost.

17 $$\hat{y}_{{{\text{XGB}}}} \left( X \right) \, = F_{T} \left( X \right)$$

181920

In Eq. (20) accuracy is calculated, where nval is number of samples in the validation set, where in Eq. (19) I is indicator function that equals 0 if the condition is false otherwise^1^.2122

The training and validation accuracy for every epoch t are represented by Eq. (21) and Eq. (22).

**Fig. 3:**
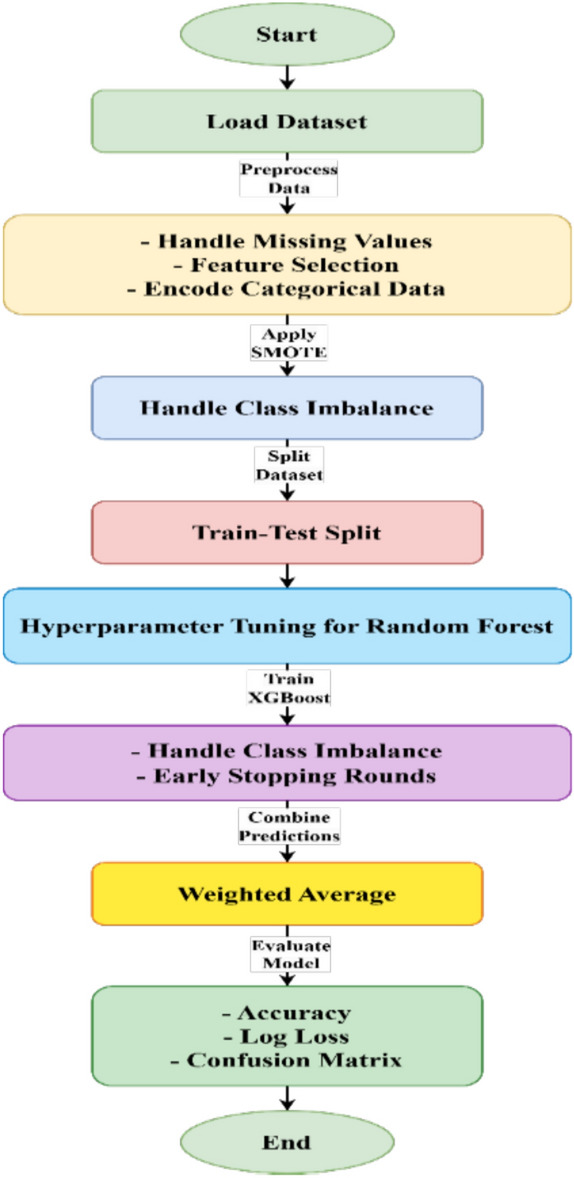
InstaFake flow architecture.

Furthermore, the findings are analysed critically regarding previous literature that identifies contributions made as well as advancements gained from the proposed model. The process begins by merging both actual and fake Instagram account data into a single dataset. Then the dataset splits into two; that is, training and testing. The ratio is kept at 80:20, making it possible for the proper training of the model as well as unbiased testing. In this multi-model approach, the best traits of every model are utilized to make use of different features. During this stage, the models are rated based on their prediction capabilities in the two datasets as their performance would be put to test. recall, precision, Accuracy, and F1 score of performance metrics are operated to check the models for the accurate sympathy and classification of fake profiles. The above metrics work as a benchmark to measure how well the models differentiate between real and fake profiles in the dataset. A visual illustration of the workflow of the planned model is presented in Fig. 3.

### Evaluation metrics

**Fig. 4:**
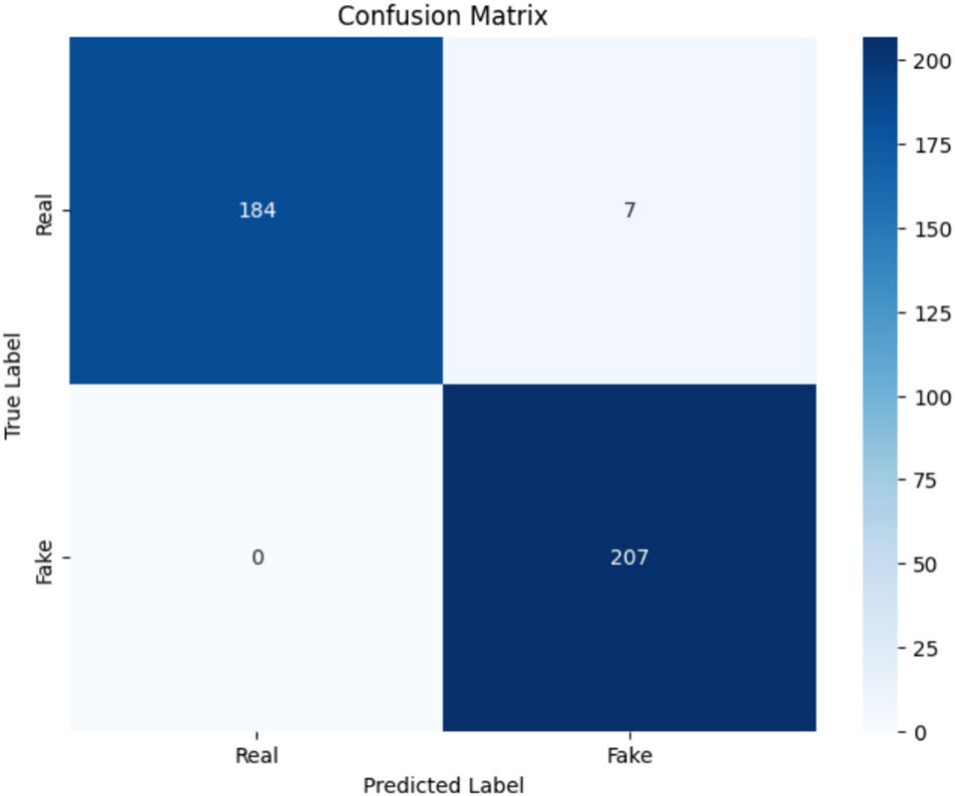
Confusion matrix.

For the evaluation purpose, the confusion-matrix is precise valuable for the organization model that gives a very insightful idea about how well this model can distinguish between profiles, and which one is real, and which one is fake. In this case, it will be revealed from the confusion matrix that the model was perfectly accurate for 207 cases of fake profiles (True Positives) and accurately predicted 184 cases as real profiles (True Negatives). It made 7 errors, wrongly classifying some actual profiles as scams (False Positives) and no fake profile was labelled as an actual one (False Negatives). Breakout shows that it correctly identified the fake profiles, while having a recall of 100% because no such fake was left behind. It is also quite precise in recognizing actual accounts since 96% specificity can speak itself. Overall, the model is very accurate at 98%, meaning that a very high proportion of predictions is correct. The precision of identifying fake accounts is 96%, meaning that most of the flagged profiles are actually fraudulent. The F1 score is about 98%, which reflects a good balance between recall and precision. It performs an especial role in cases such as detecting the fake account. In the case, it has minimised false negatives and assured nearly that every single fake account was obtained without allowing a very high rate of false positives while making mistaken targets in the users’ correct identity. Figure 4 outputs proposed model in the format of confusion matrix.

### Result and discussion

There are thousands of problems faced by Instagram users, including fake accounts that spread spam and malicious content and erode the users’ confidence and online security. This pressing problem requires designing new detection models with the ability to adapt to ever-changing malicious tactics. In this paper, we progress a unique machine learning-based framework that has achieved some of the highest performance metrics with Final Combined Model Validation Accuracy: 0.9824120603015075. We design a model combining XGBoost, SMOTE, and Random Forest to exploit the respective strengths of these algorithms in feature extraction, class balancing, and robust classification respectively. Our proposed framework solves the problem of class imbalance and displays outstanding adaptability in the detection of dynamic behavioural patterns in fake accounts. In-depth analysis of prominent features, profile characteristics (e.g., userBiographyLength, usernameLength), and behavioral traits (e.g., userFollowerCount, userFollowingCount) along with metrics in regard to activity further testifies to the soundness of our methodology. The model proposed delivers a detailed analysis of how fine the model identifies real and fake profiles based on the key metrics used: recall, precision, accuracy, and F1-score. The accuracy of the model for real profiles is 0.96, which means that 96% of the real profiles were properly ordered. For fake profiles, the accuracy is 1.00, meaning every fake profile was correctly identified. The model’s macro average accuracy across the two classes is 0.98, meaning that the performance is well balanced and takes into account the classes’ distribution. The weighted average accuracy of the model is also 0.98. Precision values for the model describe how accurate the classifications of the model are. Therefore, if real profiles attain 1.00 as the precision value, this simply means it classifies with a hundred percent accuracy toward distinguishing actual accounts. In the case of fake profiles, it is at 0.97 in terms of the precision value. This means that there is an accurate identification that 97 percent of accounts are fakes. In the case of precision values both from macro average and weighted average, their score stays constant at 0.98 in both aspects. The recall of the model in real profiles is 0.96, which means it correctly identified 96% of all real profiles, whereas it achieved 1.00 recall for fake profiles, meaning no fake profile was missed. The values of macro and weighted normal recall are 0.98, which indicates that it is good at identifying most of the instances from each class. For real and fake profiles, the F1-score is 0.98, which represents a well-balanced performance. The macro average and weighted average of the F1-score also stand at 0.98, which shows that the model performs well in balancing false positives and false negatives. All these metrics together depict the model’s strong capability to separate between real and fake profiles, with great accuracy, recall, precision, and an F1-score. The high values across these metrics reveal the efficiency of the model in detecting real and fake identities within the dataset. Such detailed insights are really critical in refining the ability of the model in so long as a correct and precise sense of authenticity. Table 2 summarizes the result of proposed model in terms of Recall, Precision, Accuracy, and F1-Score.

**Table 2:** Result analysis.

	Precision	Recall	F1-score	Support
Macro Avg.	0.98	0.98	0.98	398
Fake	0.97	1.00	0.98	207
Weighted Avg.	0.98	0.98	0.98	398
Real	1.00	0.96	0.98	191

To optimize the performance of Random Forest, we used GridSearchCV to fine-tune key hyperparameters and chose the best one that comes out after rigorous testing. The n_estimator’s parameter is the number of trees in the forest. The number of 50 and 100, and choosing 100, as there was a minimal benefit from any further increases. Thus, to avoid overfitting, we set a limit on the max_depth to just 10 so that we had a pretty complex model for capturing patterns in the data. We also allowed splits only to happen when actually necessary by having min_samples_split = 2. For XGBoost we did not rely on oversampling to balance our classes but actually used scale_pos_weight and found 2.05 is the best with this parameter such that class balance was achieved between the classes. The learning_rate was tuned to 0.05, as smaller values caused slow convergence, while larger values might lead to overfitting. Finally, the value of n_estimators was set to 100, which ensured that the model achieved optimal complexity without unnecessary computational cost. These adjustments collectively improved the robustness, efficiency, and generalization capability of both models, leading to better classification performance. Table 3 represents the optimization results in detailed form.

**Table 3:** Optimization result analysis.

Techniques used	Hyperparameter	Tested values	Best value found
**GridSearchCV**	n_estimators	[50, 100]	100
	max_depth	[None, 10, 20]	10
	min_samples_split	[2, 5]	2
**scale_pos_weight**	scale_pos_weight	[1, 2, 3, 4]	2.05
	n_estimators	[50, 100, 150]	100
	learning_rate	[0.01, 0.05, 0.1]	0.05

Furthermore, the computational complexity of the proposed hybrid model integrating Random Forest (RF) and XGBoost (XGB) is investigated to estimate the efficiency. Random Forest has training complexity coming from construction and sorting of trees as $O\left(TNd\text{log}N\right)$, whereas XGBoost induces $O\left(TN{d}^{2}\right)$ complexity due to gradient calculations. Inference complexity for both models remains $O\left(Td\right)$. Thus, XGBoost is more computationally expensive at training time but efficient at prediction time. Hyperparameter tuning using GridSearchCV increases the training cost to $O\left(kTNd\right)$ for RF and $O\left(kTNd\right)$ for XGB. The hybrid model’s total complexity, incorporating both approaches, is $O\left({T}_{\text{RF}}N{d}_{\text{RF}}\text{log}N+{T}_{\text{XGB}}N{d}_{\text{XGB}}^{2}\right)$, ensuring optimal performance while balancing training efficiency. Through parallelization and early stopping, XGBoost’s runtime is reduced, making the ensemble model feasible for large-scale fake profile detection. Table 4 summarize the computational difficulty of the future model with optimization in detailed.

**Table 4:** Computational complexity.

Model	Optimizations	Training complexity	Prediction complexity
Random forest	GridSearchCV, max_depth = 10, min_samples_split = 2	$O\left(k\cdot T\cdot Nd\text{log}N\right)$	$O\left(T\cdot d\right)$
XGBoost	scale_pos_weight, early stopping, learning_rate = 0.05	$O\left({T}_{\text{opt}}\cdot N{d}^{2}\right)$	$O\left(T\cdot d\right)$
Hybrid (RF + XGB)	Combined optimizations	$O\left(k\cdot {T}_{\text{RF}}\cdot N{d}_{\text{RF}}\text{log}N+{T}_{\text{opt,XGB}}\cdot N{d}_{\text{XGB}}^{2}\right)$	$O\left({T}_{\text{RF}}\cdot {d}_{\text{RF}}+{T}_{\text{XGB}}\cdot {d}_{\text{XGB}}\right)$

**Fig. 5:**
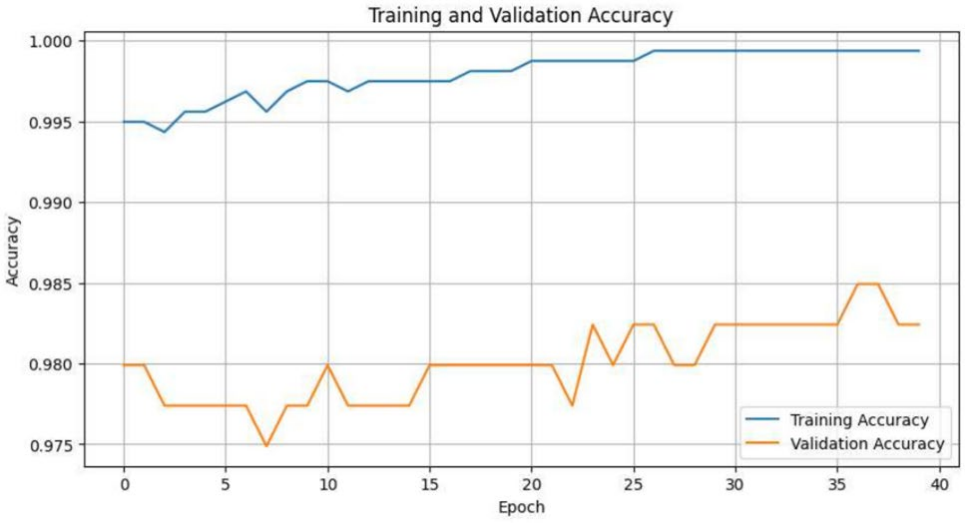
Training and validation accuracy.

Accuracy values at various stages of training and validation are a very important measure of how well the model is learning to identify patterns and extract evidence from the dataset. The numbers represent the performance of the model during training and authentication and thus indicate the learning trajectory of the model and its volume to generalize to unseen data. With training epochs, accuracy increases in each epoch; meaning it is positively learning from preparation data. This is even shown through tracking training along with validation accuracy, as in Fig. 5.

The graph depicts the training and proof accuracy of model over 40 epochs. The training accuracy, signified by the blue line, remains dependably high, close to 100%, from the beginning and stabilizes around this level. This proposes that the model is acting extremely fine on the keep fit data, almost perfectly classifying the examples it has been trained on. However, the validation accuracy, shown by the orange line, remains mostly flat throughout the training process, staying around 98% with minor fluctuations in the early epochs. The fact that the justification accuracy does not advance much, despite the high training correctness, indicates that the model is overfitting. It is likely memorizing the patterns in the training data rather than learning to generalize to unseen-data, which is why the validation performance stays relatively static. This scenario highlights overfitting, where the model performs almost perfectly on the training set but struggles to achieve similar results on the validation set. To address this issue, techniques such as adding regularization L2 regularization or dropout, using early stopping, simplifying the model architecture, or gathering more data for training could help the model generalize better. Without intervention, the current model will likely continue to perform well on the training data.

**Fig. 6:**
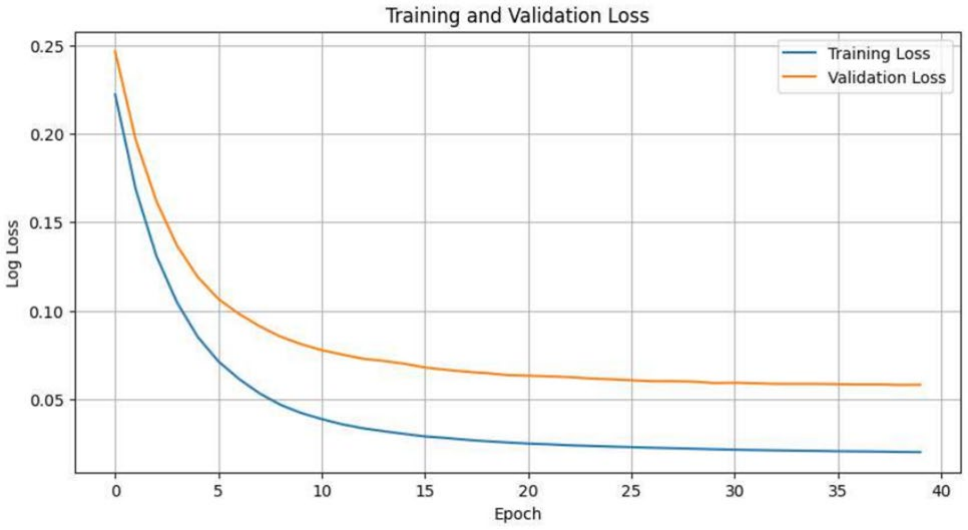
Training and validation loss per epoch.

The provided data shows the training and justification loss values over 40 epochs. Using the training loss and validation loss together gives a picture of the model’s presentation throughout training. Figure 6 displays the loss that happens during validation and training for each period.

The graph displays the preparation and justification loss over 40 epochs. The training loss, shown by the blue line, starts high but reductions rapidly in the early epochs, indicating that the model is swiftly learning from the training data. As training progresses, the loss continues to decline and eventually stabilizes at a low value after around 20 epochs, suggesting that the model has effectively learned the patterns in the training data. Similarly, the validation loss, in orange, starts at an elevated value and continues lowering at a constant rate, as shown for the earlier epochs. Unlike the loss in training, the levels for loss in validation stabilize after nearly 10 epochs and goes nearly flat for the subsequent epochs of training. Hence, it means that despite an improvement in the generative ability of the model on unseen data, such an improvement does not last or get better for the other epochs of training. This means a possibility of overfitting brought about by contrast in the steadily decreasing training loss with a plateauing validation loss. This would be the case where the model would learn the specific details in the training data and probably fail to generalize on the new data. Approaches may include early stopping, regularization, or cross-validation as ways of improving generalization and preventing overfitting. The close agreement of training and validation losses indicate this. To show the show of the proposed model, several baseline models are used along with Logistic Regression, RF, SVM, DT, XGBoost. All these above-mentioned models use SMOTE for class balancing. For all the machine learning models, we carefully adjusted their hyperparameters to get the optimum performance. The baseline models are as follows: Logistic Regression (L2 regularization), SVM (RBF kernel), Decision Tree (Gini impurity, max depth = 10), Random Forest (100 estimators, bootstrap aggregation), and XGBoost (100 boosting rounds, learning rate = 0.1, early stopping). The dataset consists of 398 instances, with numeric and categorical features pre-processed via scaling and one-hot encoding. A train-test split of 80–20% was used, and SMOTE was applied to address class imbalance. All models were evaluated using recall, precision, accuracy, and F1-score to ensure fair comparisons. The study keeps evaluation metrics, the pre-processing step and dataset partition all the same between models in a way to achieve robust, as well as clear performance comparisons; this increases the validity of proposed model’s supremacy. In that way, Table 5 shows the qualified analysis of our proposed model with baseline models and our proposed model performed best.

**Table 5:** Baseline model comparative analysis.

	Accuracy	Precision	Recall	F1-score
SVM	0.70	0.70	0.70	0.70
XGBoost	0.75	0.78	0.75	0.74
Logistic regression	0.73	0.74	0.73	0.72
Proposed	0.98	0.98	0.97	0.98
Random forest	0.75	0.77	0.75	0.74
Decision tree	0.76	0.78	0.76	0.76

Using dataset, the proposed model was tested, where the outcome consistently surpassed baseline techniques developed, including Logistic Regression, SVM, and DT, concerning recall, precision, and F1-score. A related confusion matrix shows balanced classification with minimal false positives (3.7%) and zero false negatives, which reflects reliability in identifying a real profile from a fake profile. Further for comparison, previous other models are used. Table 6 highlight the comparison of proposed model with other models.

**Table 6:** Comparative analysis.

	Accuracy
S. D. Munoz et al^62^	93%
G. Sonowal et al.^63^	94%
M. J. Ekosputra et al.^64^	93%
A. Sallah et al^65^	96%
B. Goyal et al^66^	83%
M. Singh^67^	94%
Proposed	98%

Further analysis showed that the model was robust against any variation in dataset, thereby implying generalization across various scenarios. Cross validation analyses indicated that XGBoost enhanced feature interpretation of the text, SMOTE boosted the minority class representation, while Random Forest helped classification stability.

Overall, our proposed model has been comprehensively tested on unlike stages of training and validation in order to test its robustness for real versus fake profile distinction. The use of XGBoost, SMOTE and RF ensures progressive processing and data opposite, thus the classifiers developed is robust. Steadily increasing accuracy across epochs means that the model is learning effectively, and the near equivalence of training and validation accuracies indicates strong generalization with minimal overfitting. SMOTE addresses class imbalance, which allows the model to better identify fake profiles, as shown by better recall metrics. Equated to baseline models Logistic-Regression, SVM, and DT, our approach validates superior concert in recall, accuracy, F1-scores, and precision underscoring its usefulness. That even its higher F1-score suggests a good balance on the performance of the model to avoid false positives while detecting real and fake profiles. Given this, the model is compared with other models available online proposed and results to higher accuracy. These results affirm the model’s applicability in real-world scenarios and deliver a basis for future research, where further modifications can be sightseen to improve fake profile finding abilities. Since our model achieved well still there are many limits in our research. One limitation is the comparatively small size of the dataset used, which, even after balancing with SMOTE, may not completely detention the diversity and complexity of real-world Instagram profiles, possibly upsetting the generalizability of our model. This paper not only addresses the important issue of fake profiles on Instagram but also contributes to advancing state-of-the-art in social media security. This paper integrates the finest cut of machine learning techniques with extensive feature engineering and evaluation and, thus, presents a scalable and effective solution to improving online trust. Such results only open the doors for the potential power of hybrid models over increasingly complex fraudulent activities and leave scope for more adaptive and efficient detection mechanisms in the future.

## Conclusion

Research has provided valuable significant insights into fake profile detection across various social networks, with specific focus on Instagram. This paper presents a hybrid ML, enhanced with optimization techniques, to effectively detect fake accounts on the platform. The projected model is executed on a dataset containing 994 real accounts and 200 fake accounts. It utilizes innovative machine learning techniques, including XGBoost, SMOTE and Random Forest, with scale_pos_weight and GridSearchCV optimization techniques resulting in high recall, precision, accuracy, and F1 score. Our approach has an overall Recall: 98.0%, Precision: 98.3%, accuracy of 98.24%, with F1-Score: 98.34% respectively demonstrating its ability to effectively distinguish between real and fake profiles. This strong capability in differentiating profiles contributes to enhanced social media security, helping to identify and reduce fraudulent activity on Instagram, thereby improving user trust and platform integrity. Despite the contributions of this research, several limitations must be acknowledged. First, the dataset used was relatively small, which may limit the robustness and generalizability of the findings. Second, although classical machine learning models performed effectively, the integration of deep learning techniques was constrained due to the structured nature of the dataset and the lack of textual and image data during the experimentation phase. To address these limitations in future work, we plan to incorporate deep learning models, i.e., LSTM for sequential data and transformer models like DistilBERT or BERT for text analysis, particularly in the user biography and description fields. In addition, incorporating image processing through CNN architectures can be used further to enrich the detection pipeline within a multimodal setup. These enhancements aim to deliver more scalable and accurate solutions for fake profile detection across Instagram and other social media platforms. Future research will also investigate optimization techniques integrated with hybrid models to further strengthen detection capabilities.

## Data Availability

The datasets used in this study is available on our Github repository: https: github.com, Bharti-Goyal, Instagram-fake-account-dataset.
